# The Impact of the *NOD2/CARD15* Variant (3020insC) and *PSMA6* Polymorphism (-8C>G) on the Development and Outcome of Multiple Myeloma

**DOI:** 10.1155/2020/7629456

**Published:** 2020-06-06

**Authors:** S. Zmorzyński, S. Popek-Marciniec, W. Styk, M. Wojcierowska-Litwin, I. Korszeń-Pilecka, A. Szudy-Szczyrek, S. Chocholska, M. Hus, A. A. Filip

**Affiliations:** ^1^Department of Cancer Genetics with Cytogenetic Laboratory, Medical University of Lublin, Poland; ^2^Institute of Psychology, The John Paul II Catholic University of Lublin, Lublin, Poland; ^3^Chair and Department of Hematooncology and Bone Marrow Transplantation, Medical University of Lublin, Poland

## Abstract

**Introduction:**

Multiple myeloma (MM) is a hematological malignancy characterized by genetic variety. The 3020insC variant of the *NOD2/CARD15* gene results in the upregulation of proinflammatory cytokines. Chronic inflammation and abnormal function of the proteasome system may lead to MM development. The polymorphism (-8C>G) in the *PSMA6* gene affects proteasome activity. The aim of our study was to analyze the possible relationship of *NOD/CARD15* and *PSMA6* genes with the risk of development and outcome of MM, as well as the sensitivity to bortezomib (proteasome inhibitor) in cell cultures derived from MM patients. *Objects and Methods*. Genomic DNA from 100 newly diagnosed MM patients and 100 healthy blood donors was analyzed by methods such as PCR-RFLP (for *PSMA6* genotyping) and automated DNA sequencing (for *NOD2/CARD15* genotyping). In a subgroup of 50 MM patients, nucleated bone marrow cells were treated with bortezomib *in vitro*.

**Results:**

Patients with *PSMA6* CG+GG genotypes had higher chances for progressive disease (OR = 5.0, 95% CI 1.07-23.16, *p* = 0.05), shorter overall survival taking into account the type of treatment (*p* = 0.039), and increased risk of death due to MM at the level of tendency (OR = 4.74, 95% CI 1.02-21.97, *p* = 0.06). The presence of *NOD2/CARD15* 3020insC decreased the risk of renal dysfunction in MM (OR = 0.23, 95% CI 0.07-0.74, *p* = 0.009). The analyzed changes in *NOD2/CARD15* and *PSMA6* genes did not impact the MM risk. In an *in vitro* study, bortezomib increased the number of apoptotic cells at 8 nM and 12 nM between wild-type and 3030insC variants of *NOD2/CARD15* (*p* = 0.018 and *p* = 0.03, respectively).

**Conclusion:**

The presented results suggest a possible impact of *PSMA6* CG+GG genotypes on the MM outcome and the association of the *NOD2/CARD15* variant with bortezomib *in vitro* sensitivity.

## 1. Introduction

Multiple myeloma (MM) is a hematological, heterogeneous malignancy characterized by infiltration of bone marrow by clonal plasma cells [1]. Inflammatory mediators, such as nuclear factor *κ*B (NF-*κ*B), are critical in the malignant transformation [2, 3]. Chronic inflammation with elevated NF-*κ*B can support the development of hematological malignancies, including MM [4]. Activation of this pathway is enhanced by nucleotide oligomerization domain 2 (NOD2 also known as CARD15) [5]. This protein is encoded by the *NOD2/CARD15* gene (*locus* 16q12.1), is a component of the host defence against intracellular pathogens, and plays an important role in immune system function. The *NOD2/CARD15* gene consists of 18 exons, and its expression is observed in monocytes, macrophages, granulocytes, and some epithelial cells [5, 6]. The most common variant in the *NOD2/CARD15* gene is present in the form of 3020insC, which leads to a stop codon in exon 11. The result of a nonsense change is the production of truncated NOD2 protein with the last 33 amino acids missing (Leu1007fsinsC) [5]. The variant leads to loss-of-gene function, and truncated protein has a reduced ability to recognize bacterial muramyl dipeptide and consequently to stimulate NF-*κ*B response [5, 7]. On the other hand, the absence of wild-type NOD2 protein results in the upregulation of proinflammatory cytokines [8]. The 3020insC variant of *NOD2/CARD15* also predisposes cells to many types of common cancers, mainly solid tumors—for example, ovarian cancer, breast cancer, and colorectal cancer [7, 9, 10].

The processes like apoptosis, proliferation, and inflammation are controlled in a nondirective way by the proteasome system [11]. It is a multicatalytic proteinase complex. The 20S proteasome is composed of seven *α*-subunits and ten *β*-subunits. The human proteasome subunit *α*6 (or p27K) is encoded by the *PSMA6* gene (*locus* 14q13), which contains single-nucleotide polymorphism (SNP) in 5′-UTR (rs10489900). This SNP is caused by substitution (-8C>G) where nucleotide with cytosine (C allele) is replaced by guanine (G allele). The G allele is associated with a higher transcription rate of the *PSMA6* gene in human B cells in comparison to the C allele [12]. The proteasome-dependent pathway and higher expression of the *PSMA6* gene may activate NF-*κ*B, which leads to inflammation and transformation to MM [13].

The proteasome system consists of the 26S proteasome (including two subunits of 20S and 19S) and immunoproteasome [14]. The 26S proteasome is involved in the degradation of proteins regulating the cell cycle, cell migration, proliferation, angiogenesis, and apoptosis [15]. These proteins play a role in malignant transformation. Therefore, proteasome inhibitors preventing angiogenesis and cell migration and decreasing inflammatory response have been developed and tested for antitumor response [14, 15]. In MM patients, significant improvements in survival have been obtained, among others, by the introduction of bortezomib, a 26S proteasome inhibitor, as well as an anti-inflammatory agent—thalidomide [16]. Inhibition of the proteasome pathway in cancer cells leads to apoptosis via the activation of unfolded or abnormal protein response [17].

Taking the above into account, we hypothesized that variants in the *NOD2/CARD15* gene and *PMSA6* polymorphism may affect the development and outcome of MM. In this paper, we present an analysis for the studied genetic variants individually and in different combinations to evaluate the role of these genes in the MM susceptibility, survival, and response to treatment with thalidomide and/or bortezomib. Furthermore, the aim of our research was to analyze the studied DNA changes in the sensitivity prediction in cell cultures derived from MM patients. To our knowledge, the presented results were not previously published by other authors.

## 2. Material and Methods

### 2.1. Patients and Samples

For the study, bone marrow aspirates and peripheral blood samples were collected from 100 newly diagnosed patients with MM (with mean age 65.32 years), who were hospitalized at the Chair and Department of Hematooncology and Bone Marrow Transplantation, Medical University of Lublin, in years 2013-2019. The study obtained positive opinions from the Bioethics Committee of the Medical University of Lublin (nos. KE-0254/165/2013 and KE-0254/337/2016), according to the ethical standards established by the Helsinki Declaration. The research material was collected upon all patients (*n* = 100) and healthy blood donors (*n* = 100) who provided written informed consent.

Control samples were made of peripheral blood obtained from 100 healthy blood donors (50 males/50 females, with mean age 34.4 years) attending the Regional Blood Donation and Blood Treatment Center in Kielce, Poland.

The general characteristics of MM patients are shown in Table 1. The inclusion and exclusion criteria are described in Table 2.

Therapeutic induction regimens consisted of thalidomide and/or bortezomib combined with steroids and/or cyclophosphamide. 37 MM patients underwent autologous hematopoietic stem cell transplantation (auto-HSCT). Response to treatment was evaluated according to the International Myeloma Working Group guidelines and classified as stringent complete remission (sCR), complete response (CR), very good partial response (VGPR), partial response (PR), minimal response (MR), stable disease (SD), or progressive disease (PD) as described elsewhere [18, 19]. Overall survival (OS) encompassed time from diagnosis until relapse, progression, death due to a tumor effect, or the last follow-up and time from diagnosis until death by any cause or the last follow-up. The median follow-up time of MM patients enrolled in the study was 18 months. Progression-free survival (PFS) was estimated as the time elapsed between treatment initiation and tumor progression or death from any cause [20].

Peripheral blood was used for DNA isolation and studied genetic variant determination.

Cell cultures were established from bone marrow aspirates to carry out the research associated with cIg-FISH (*n* = 100) and bortezomib treatment (*n* = 50). In an *in vitro* bortezomib study, MM patients without chromosomal aberrations were included.

### 2.2. DNA Isolation

DNA isolation from peripheral blood was performed using a commercial kit (Qiagen, Germany) according to the manufacturer's procedure. The concentration and quality of DNA were checked using a NanoDrop device (Thermo Fisher Scientific, USA).

### 2.3. *PSMA6* Genotyping

For the analysis of the *PSMA6* polymorphism, the PCR restriction fragment length polymorphism (PCR-RFLP) method was applied according to the validated protocol of Bachmann et al. [21]. A *PSMA6* gene fragment length of 100 bp was amplified by PCR using the following primers:

- Forward 5′-CTG GTG CGG GAG CTA CGG G-3′
- Reverse 5′-AAT GGT AAT GTG GCG GTC AAA AC-3′

Each PCR mixture (25 *μ*l) contained 100 ng genomic DNA and PCR buffer (Clontech), dNTP mixture (0.25 mM), HD polymerase (Clontech), and primers (10 *μ*M of each). The touchdown PCR method was used. The mixture was heated at 95°C for 5 min and underwent 14 cycles of amplification: denaturation at 95°C for 30 s, annealing at 64.5°C for 20 s (-0.5°C/per cycle), and elongation at 72°C for 20 s. After 14 cycles, the mixture underwent 20 cycles with a constant temperature of 57.5°C. The denaturation and elongation temperatures and times were the same as the above. The final elongation takes 5 min at 72°C. The PCR was performed in the Applied Biosystems 9700 Thermal Cycler.

**Figure 1:**
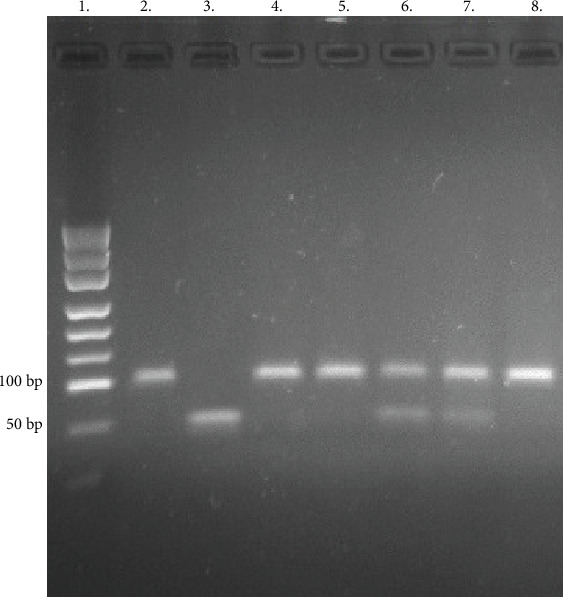
PCR-RFLP of *PSMA6* gene polymorphisms. Lane 1—ladder (34, 67, 110, 147, 190, 242 331, 404, and 504 bp). Lanes 2, 4, 5, and 8—CC genotypes. Lanes 6 and 7—CG heterozygotes. Lane 3—GG homozygote.

The PCR product was digested with *RsaI* (Thermo Fisher Scientific) for 16 hours at 37°C producing two fragments of 50 bp or one fragment of 100 bp for the presence of the G or C allele, respectively. RFLP products were analyzed on 3% agarose gel and stained with SimplySafe (EURx, Poland) and visualized in G:Box (Syngene, Great Britain) (Figure 1). An independent PCR analysis was carried out for each sample.

### 2.4. *NOD2/CARD15* Genotyping

The *NOD2/CARD15* frameshift change (3020insC) was analyzed by the use of automated DNA sequencing. The following primers were used to amplify 533 bp:

- Forward 5′-CTG AGC CTT TGT TGA TGA GC -3′
- Reverse 5′-TCT TCA ACC ACA TCC CCA TT-3′

**Figure 2:**
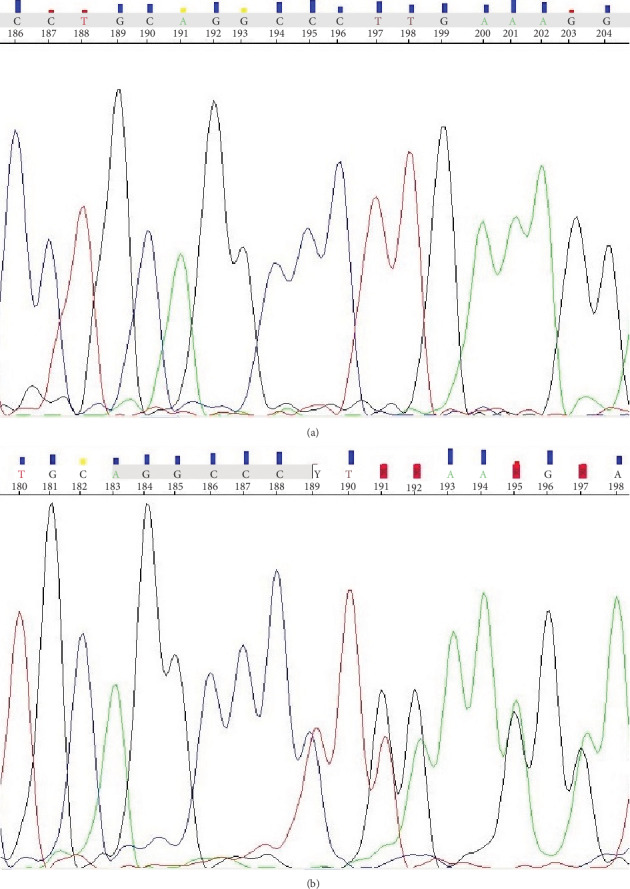
Electropherograms of the *NOD2/CARD15* gene obtained by automated Sanger DNA sequencing: (a) wild-type sequence and (b) insertion of C at 3020 nucleotides (in this case 189 nucleotides).

Each PCR mixture (25 *μ*l) contained 50 ng genomic DNA, PCR buffer (Clontech), dNTP mixture (0.25 mM), HD polymerase (0.31 U) (Clontech), and primers (10 *μ*M of each). The mixture was heated at 94°C for 5 min and underwent 35 cycles of amplification: denaturation at 98°C for 15 s, annealing at 60°C for 10 s, and elongation at 72°C for 20 s. The final elongation takes 5 min at 72°C. The PCR was performed in the Applied Biosystems 9700 Thermal Cycler using the HD Advantage polymerase (Clontech). Sequencing PCR was performed with the use of the BigDye Terminator v3.1 Cycle Sequencing Kit (Applied Biosystems) in a thermal cycler (as described previously). The sequencing PCR product was purified by the use of an exterminator kit (A&A Biotechnology). The sequencing run module was StdSeq50_POP7 in genetic analyzer 3130 (Applied Biosystems). The results were analyzed by the use of Applied Biosystems software (Figure 2).

### 2.5. Cytogenetic Analyses

Abnormalities essential for MM, such as del(17p13.1) and *IgHV* gene rearrangements—t(4;14) and t(14;16)—were tested by simultaneous staining of cytoplasmic immunoglobulin and FISH (cIg-FISH) according to Ross et al.'s recommendations and were identified according to the previously described protocol with modifications [20–24].

### 2.6. Bortezomib *In Vitro* Treatment

Bone marrow aspirates (*n* = 50) (mean number of plasma cells—31.31% ± 20.69) were used to establish cell cultures as described by Zmorzynski et al. [20]. The number of apoptotic, necrotic, and viable cells was determined by means of the Annexin V-Cy3 Apoptosis Detection Kit according to the manufacturer's protocol (Sigma-Aldrich, USA) and following the methods of Zmorzynski et al. [24].

### 2.7. Statistical Analysis

Laboratory values of MM patients with polymorphisms were compared using an independent *t*-test for continuous variables and the chi-square test for categorical variables. The association of the studied polymorphisms with prognostic factors was evaluated using the chi-square test or Fisher's exact test (when one expected value was <5). The quantitative data was shown as frequency or percentage. Deviation of genotype frequencies in controls and cases from the Hardy-Weinberg equilibrium (HWE) was assessed by the chi-squared test with Yates's correction for the groups with less than five patients [25]. For 95% confidence interval (CI), we assumed *p* = 0.05 and *χ*^2^ = 3.84; therefore, if *χ*^2^ ≤ 3.84 and the corresponding *p* ≥ 0.05, then the population is in HWE. The logistic regression was used to evaluate the fold risk of MM. The Cox proportional hazard model was used for univariate and multivariate analyses of OS and PFS. Variables of the international staging system (ISS), auto-HSCT, and genotypes (without combinations) were included in multivariate analysis. The Kaplan-Meier method and the log-rank test were used for survival analysis. The statistical power of the study was calculated according to Bacchetti and Leung [26]. We assumed a 5% error of inference and the related level of significance *p* < 0.05 pointing to the existence of statistically significant differences. Statistical analyses were performed using the Statistica ver. 12.5 (StatSoft) software.

## 3. Results

The presented study included 100 MM patients, 53 males and 47 females, with a median age of 65.32 years. Genotyping was successful in all the individuals investigated within the study. The HWE test confirmed that the genotypic frequencies of *PSMA6* and *NOD2/CARD15* genes for healthy individuals (controls) and MM patients were balanced (Table 3). The allelic frequencies of the *PSMA6* or *NOD2/CARD15* gene between study and control groups were statistically insignificant (Table 4). The studied *PSMA6* and *NOD2/CARD15* variants did not impact on the risk of MM (Table 5). The CG and GG genotypes of the *PSMA6* gene were analyzed together, because the number of GG cases was very low (Table 3). Moreover, we analyzed the effect of both changes—3020insC of the *NOD2/CARD15* gene and CG+GG genotypes of the *PSMA6* polymorphism—on the risk of MM, which was statistically insignificant (Table 5). In the case of the 3020insC variant of the *NOD2/CARD15* gene, only heterozygotes were observed.

A univariate Cox analysis revealed that patients at stage III according to ISS had a 1.7-fold increased risk of death (*p* = 0.003) (Table 6). Similar findings were observed in the case of disease relapse or progression in MM patients at stage III (HR = 1.68, *p* < 0.001) and without auto-HSCT (HR = 2.54, *p* = 0.002) (Table 6). Moreover, the univariate Cox analysis did not show an increased risk of death or disease relapse/progression in MM patients with the *PSMA6* polymorphism and/or *NOD2/CARD15* variant. The multivariate Cox regression analysis confirmed that patients without auto-HSCT or at stage III according to ISS had an increased risk of death or disease relapse/progression, respectively (Table 7).

The analysis of the response rate in MM showed that patients at stage III or without auto-HSCT had an increased chance of progressive disease (PD) (Table 8). Moreover, patients with CG+GG genotypes of *PSMA6* had higher chances for PD (Table 8). The statistical power of this association was 0.63. The CG+GG genotypes had an increased risk of death due to MM at the level of tendency (OR = 4.74, 95% CI 1.02-21.97, *p* = 0.06).

Furthermore, we analyzed the potential relationships between clinical and laboratory results and selected genotypes. We found that the *NOD2/CARD15* variant decreased the risk of renal dysfunction in MM patients (OR = 0.23, 95% CI 0.07-0.74, *p* = 0.009). We did not confirm any significant correlations among the studied variants of *NOD2/CARD15* and *PSMA6* genes regarding clinical parameters (Table 9). The odds ratio was used to analyze the relationship of the *PSMA6* polymorphism and *NOD2/CARD15* variant with chromosomal aberrations in MM patients. The results were statistically insignificant for *PSMA6* CG+GG (OR = 1.22, 95% CI 0.36-4.13, *p* = 0.97), as well as for the *NOD2/CARD15* variant (OR = 2.63, 95% CI 0.55-12.52, *p* = 0.34). Taking into account the type of chromosomal aberration and studied genotypes, we did not find statistically significant results - *PSMA6* CC versus CG+GG - del(17p13.1) (*p* = 0.11), t(4;14) (*p* = 0.64), t(14;16) (*p* = 0.89) and *NOD2*/*CARD15* wild type versus 3020insC variant - del(17p13.1) (*p* = 0.56), t(4;14) (*p* = 0.93), t(14;16) (*p* = 0.72).

**Figure 3:**
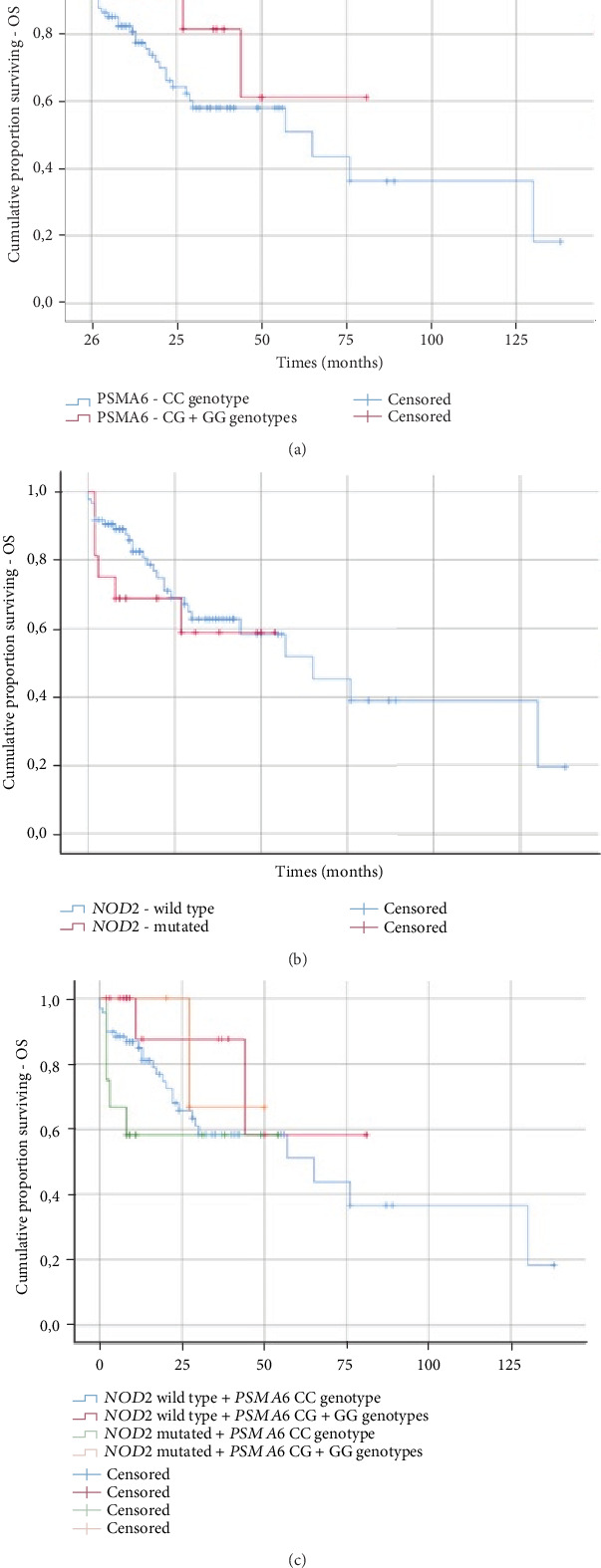
Kaplan-Meier analysis of OS in the group of MM patients with (a) *PSMA6* genotypes (log-rank test *p* = 0.142), (b) *NOD2/CARD15* genotypes (log-rank test *p* = 0.487), and (c) *PSMA6* and *NOD2/CARD15* genotypes (log-rank test *p* = 0.367).

**Figure 4:**
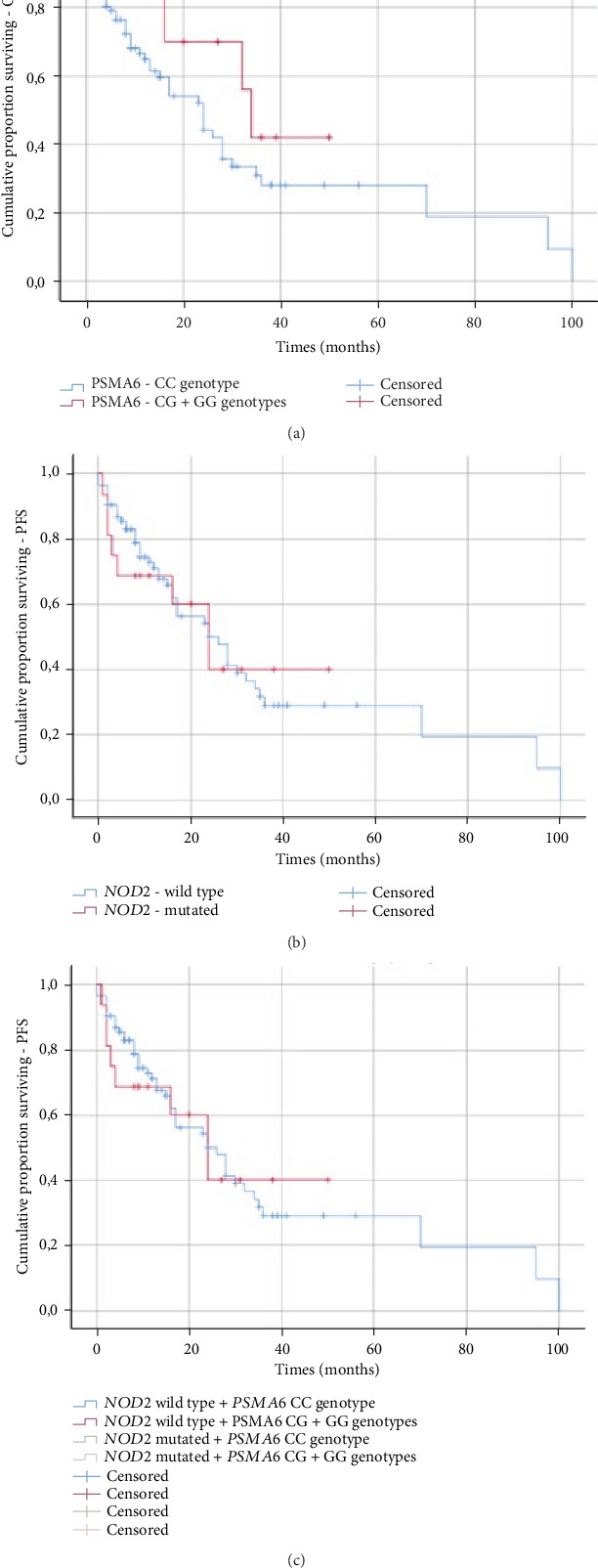
Kaplan-Meier analysis of PFS in the group of MM patients with (a) *PSMA6* genotypes (log-rank test *p* = 0.059), (b) *NOD2/CARD15* genotypes (log-rank test *p* = 0.937), and (c) *PSMA6* and *NOD2/CARD15* genotypes (log-rank test *p* = 0.244).

We analyzed (by ANOVA) the association between the studied genotypes and survival of MM patients. Without taking into account the treatment, we did not find statistically shorter OS and PFS in patients with the wild-type *NOD2*/*CARD15* or *PSMA6* CC genotype in comparison to those with the 3020insC variant of *NOD2*/*CARD15* or *PSMA6* CG+GG genotypes (Table 9). In the log-rank test (Figures 3 and 4) without taking into account the type of treatment, the tendency for shorter PFS in patients with CG+GG genotypes was observed (Figure 4). Furthermore, a log-rank analysis taking into account the studied changes and the types of treatment (thalidomide versus bortezomib versus both—thalidomide and bortezomib) was performed. The *NOD2/CARD15* 3020insC variant did not impact the type of treatment versus OS (*p* = 0.213) or PFS (*p* = 0.122). Similar results were observed in the case of the CC genotype of the *PSMA6* gene (OS (*p* = 0.182) and PFS (*p* = 0.095)). Individuals with CG+GG genotypes treated with bortezomib (VCD) or both—bortezomib and thalidomide (VTD)—had shorter OS in comparison to those treated with thalidomide (CTD) (*p* = 0.039). Shorter PFS (at the level of tendency) was observed in patients with CG+GG genotypes treated with bortezomib (VCD) or both (bortezomib and thalidomide (VTD)) in comparison to those treated with thalidomide (CTD) (*p* = 0.06).

In *in vitro* studies, bortezomib increased the number of apoptotic and necrotic cells in all the studied genotypes. The only statistically significant differences were observed in the number of apoptotic cells at concentrations of bortezomib at 8 nM and 12 nM between wild-type and 3020insC variants of *NOD2*/*CARD15* (Table 10).

## 4. Discussion

To our knowledge, this is the first study elucidating the correlation of the *PSMA6* polymorphism and *NOD2*/*CARD15* variant with the risk and the outcome of MM, as well as the response to bortezomib under *in vitro* conditions.

Little is known about the role of the *PSMA6* -8C>G polymorphism in cancer development, including MM. Most of the research was focused on the association of this SNP with the risk of cardiovascular disease and diabetes mellitus [27, 28]. Our findings suggest that CG+GG genotypes of the *PSMA6* gene might be associated with the response rate of MM patients and shorter OS taking into account the type of treatment. The G allele of this SNP leads to higher promoter activity and elevated *PSMA6* gene expression in human B cells in comparison to the C allele [12]. Higher proteasome activity, as a result of the GG genotype, may lead to degradation of misfolded or unfit antiapoptotic proteins [29]. Bachmann and coworkers in 116 MM patients showed shorter median survival time for individuals with the GG genotype [21]. Moreover, they revealed in Cox multivariate analysis that the G allele was an independent prognostic factor for MM patients [21]. In our Cox multivariate analysis, we did not confirm the association of the G allele with the MM outcome. However, in the log-rank test, we observed higher chances of progression in patients with CG+GG genotypes. Additionally, taking into account the type of treatment, we found shorter OS in individuals with CG+GG genotypes treated with bortezomib/bortezomib+thalidomide in comparison to those (with CG+GG genotypes too) treated with thalidomide. It has a different mechanism of action in comparison to bortezomib. Thalidomide, as an immunomodulatory drug, inhibits the NF-*κ*B pathway and provides better survival of MM patients [30]. The inflammation development through activation of the NF-*κ*B pathway may lead to malignant transformation, as well as disease progression [13].

The *α*6 subunit, encoded by the *PSMA6* gene, is present in the core of the 20S proteasome. It belongs to proteasome-interacting proteins and among others with the *β*5 subunit plays a role as an auxiliary factor. Suppression of the proteasome leads to endoplasmic reticulum stress and apoptosis [31]. Inhibition of the proteasome using *β*5 subunit blockers like bortezomib results in cell cycle arrest and induction of apoptosis in MM cells [32]. However, in our study, we did not observe the relationship between the *PSMA6* gene polymorphism and response to bortezomib treatment under *in vitro* conditions. It is possible that the *α*6 subunit in comparison to *β*5 of the 20S proteasome does not modify substantially the proteasome function.

The change in the *NOD2*/*CARD15* gene in the form of C insertion in a region encoding the ligand recognition domain (exon 11) is described as polymorphism (rs2066847) or mutation [5, 9, 10, 33, 34]. The difference is due to the fact that in cancer research, it is called mutation and in a noncancer field, the term polymorphism is used. The current guidelines of the American College of Medical Genetics and Genomics and the Association for Molecular Pathology recommend the use of the neutral term “variant” only [35]. The 3020insC variant of the *NOD2*/*CARD15* gene is implicated in the development of chronic inflammation and increased risk of solid tumors such as colorectal, gastric, breast, and lung cancer, as well as non-Hodgkin's lymphoma [10, 36–39]. Kurzawski et al. found a statistically significant difference in mutated *NOD2*/*CARD15* allele distribution in 250 nonhereditary nonpolyposis colorectal cancer patients in comparison to healthy individuals [36]. In our study, we did not observe differences in *NOD2*/*CARD15* wild-type/3020insC allele distribution between MM patients and the control group. The frequency of these alleles in our study populations was in HWE. Moreover, we did not find a statistically significant impact of the *NOD2/CARD15* variant on OS and PFS of MM patients. This result is consistent with the observation of Huszno and coworkers. In their study in breast cancer patients, they found no difference in OS between 3020insC variant carriers and wild-type homozygotes of the *NOD2*/*CARD15* gene [9].

Furthermore, we found that the *NOD2/CARD15* variant decreased the risk of renal dysfunction in MM patients, which was not previously reported by other researchers. In an animal model, Stroo and coworkers observed no difference in renal injury between wild-type *Nod2* double-knockout mice with chronic kidney disease [40]. They concluded that *Nod2* does not play an important role in the development of renal damage [40]. On the other hand, Kruger et al. observed an unfavorable effect of the *NOD2/CARD15* wild type on the outcome of graft survival in 352 patients receiving their first renal transplant [41]. Moreover, they took into account two additional SNPs (SNP8—R702W and SNP12—G908R) present in the *NOD2/CARD15* gene, which are associated with increased susceptibility to Crohn's disease [41]. It is possible that the *NOD2/CARD15* variant showed only a minor effect and cooccurrence of these SNPs may affect or modify the function of NOD2 protein. In our further research, it would be worth extending the analysis by these two SNPs.

In our *in vitro* study, the 3020insC variant of the *NOD2*/*CARD15* gene was associated with statistically significant lower levels of apoptotic cells at 8 nM and 12 nM of bortezomib. The loss of function of the *NOD2/CARD15* gene in MM leads to apoptosis inhibition. It is known that overexpression of *NOD2/CARD15* enhances apoptosis via caspase-9 [42]. It would be desirable to examine the *NOD2/CARD15* gene expression in plasma cells after *in vitro* bortezomib treatment.

There are some limitations of our study associated with the number of MM patients recruited to the study, as well as the used methods.

The sample size was relatively small, in part due to the low incidence of the disease. We have collected detailed clinical data for 100 MM patients. Some patients were not included into this analysis, because they are still in follow-up and their clinical data are incomplete. However, in our research, the number of 100 MM patients is enough for most analyses. Some of them were not possible as a result of low frequency of genotypes. Further analysis on a larger cohort can help better understand the significance of the studied changes in the pathobiology of MM, especially in the case of disease outcome.

In our *in vitro* study, we used RPMI media with 10% FCS. Currently, AIM-V media are used instead of RMPI and FCS as they induce apoptosis of primary B cells [43]. In our control samples without bortezomib, spontaneous apoptosis was observed, and therefore, in our further research, apoptosis evaluation will be done using serum-free AIM-V medium. Moreover, for the evaluation of apoptosis, fluorescent microscopy instead of flow cytometry-based apoptosis detection (FACS) was used. FACS analysis is more reliable and quantitative for apoptosis evaluation. Unfortunately, during the experiment time, the flow cytometry method was not available for us and retrospective analysis of apoptosis is not possible. The set used for apoptosis and necrosis detection was dedicated and validated to fluorescent microscopy.

In this paper, we analyzed, for the first time, the significance of the *PSMA6* polymorphism and *NOD2*/*CARD15* variant in the survival and response to therapy of MM patients. In conclusion, these data underscore the possible relationship between *PSMA6* CG+GG genotypes and response rate of MM patients, as well as shorter OS taking into account the type of treatment. Moreover, we observed an association of the *NOD2/CARD15* variant with the lower risk of renal dysfunction in the study group and lower apoptosis levels in cell culture with bortezomib.

## Figures and Tables

**Table 1:** The characteristics of MM patients included to the study.

Variables	MM patients (*n* = 100)
Sex	
Male	53
Female	47
Type of MM^∗^	
IgG	56
IgA	25
Light chain	19
Stage according to the international staging system^∗^	
I	28
II	30
III	42
Renal failure^∗^	
No	82
Yes	18
The stage of chronic kidney disease (grade)	
G1	30
G2	28
G3A	16
G3B	12
G4	7
G5	7
Cytogenetic changes^∗^	
del(17p13.1)	14
t(4;14)	15
t(14;16)	2
Anemia grade before treatment (WHO)	
Absent	28
I—mild	32
II—moderate	30
III—severe	10
Chemotherapy	
Cyclophosphamide, thalidomide, dexamethasone (CTD)	49
Velcade, cyclophosphamide, dexamethasone (VCD)	29
Velcade, thalidomide, dexamethasone (VTD)	22

^∗^At diagnosis. M: mean; SD: standard deviation.

**Table 2:** Inclusion and exclusion criteria for MM patients and blood donors.

Inclusion criteria	
MM patients	(i) Newly diagnosed MM patients who are required to start active treatment according to the IMWG published in 2014 (ii) Signed informed consent (iii) Years after 18 (iv) Patients must have measurable disease, defined as follows: (a) For secretory MM, measurable disease is defined as the presence of quantifiable monoclonal component (≥0.5 g/dl) (b) For poor secretory or nonsecretory MM, the level of the affected serum free light chain must be ≥10 mg/dl (≥100 mg/l, with an abnormal free light chain ratio) (v) Eastern Cooperative Oncology Group (ECOG) performance status ≤ 3 (vi) Life expectancy more than 3 months
Control group	(i) Age: 18 and older (ii) Signed informed consent

Exclusion criteria	
MM patients	(i) Active smoldering MM (ii) Active plasma cell leukemia (iii) Documented systemic amyloid light chain amyloidosis (iv) Active central nervous system involvement with MM (v) Active other hematologic malignancies or solid tumors
Control group	(i) Known to be infected with HIV, syphilis, tuberculosis, hepatitis B, or hepatitis C (ii) A condition in which repeated blood draws or injections pose more than minimal risk for the subject such as hemophilia, other severe coagulation disorders, or significantly impaired venous access (iii) A condition that requires active medical intervention or monitoring to avert serious danger to the participant's health or well-being

**Table 3:** Hardy-Weinberg equilibrium (HWE) for *PSMA6* and *NOD2/CARD15* variants in the case and control groups according to expected (E) and observed (O) values.

Groups	Genotypes			Total	HWE *p* value and *χ*^2∗^
*PSMA6* gene					
—	CC	CG	GG	—	—
Control					
E	84.64	14.72	0.64	100	*p* = 0.39, *χ*^2^ = 0.71
O	84	16	0	100	
Case					
E	81	18	1	100	*p* = 1.0, *χ*^2^ = 0
O	81	18	1	100	
*NOD2/CARD15* gene					
—	Wild-type homozygotes	Heterozygotes	3020insC homozygotes	—	—
Control					
E	96.04	3.92	0.04	100	*p* = 0.29, *χ*^2^ = 0.58
O	96	4	0	100	
Case					
E	92.16	7.68	0.16	100	*p* = 0.23, *χ*^2^ = 0.63
O	92	8	0	100	

^∗^If *χ*^2^ ≤ 3.84 and the corresponding *p* ≥ 0.05, then the population is in HWE.

**Table 4:** The comparison of *PSMA6* and *NOD2/CARD15* allele frequencies among MM patients and controls.

Alleles	MM, *n* of alleles (%)	Controls, *n* of alleles (%)	*p* values
*PMSA6*			
C	180 (90)	184 (92)	0.58
G	20 (10)	16 (8)	
Total	200 (100)	200 (100)	—
*NOD2/CARD15*			
Wild type	192 (96)	196 (98)	0.24
Mutated	8 (4)	4 (2)	
Total	200 (100)	200 (100)	—

**Table 5:** Comparison of the impact of the *NOD2/CARD15* mutation and *PSMA6* polymorphism on the risk of MM.

Genotypes		MM patients	Controls	OR	95% CI	*p* value
*NOD2/CARD15*						
Wild type		92	96	Reference	—	—
Mutated		8	4	0.48	0.14-1.64	0.23
*PMSA6*						
CC		81	84	Reference	—	
CG+GG		19	16	0.81	0.39-1.68	0.57
*NOD2/CARD15*	*PSMA6*					
Wild type	CC	69	78	Reference	—	—
Mutation	CG+GG	4	1	0.22	0.02-2.02	0.31

**Table 6:** Univariate Cox analysis in the survival of MM patients.

Variable	Univariate Cox analysis for OS			Univariate Cox analysis for PFS		
	*p* value	HR	95% CI	*p* value	HR	95% CI
ISS						
I+II	—	R	—	—	R	—
III	0.003	1.69	1.18-2.41	<0.001	1.68	1.27-2.22
Auto-HSCT						
Yes	—	R	—	—	R	—
No	<0.001	5.30	2.11-13.34	0.002	2.54	1.37-4.70
*PSMA6*						
CC	—	R	—	—	R	—
CG+GG	0.13	0.43	0.14-1.31	0.33	1.15	0.15-3.96
*NOD2/CARD15*						
Wild type	—	R	—	—	R	—
Mutated	0.49	0.73	0.29-1.78	0.93	0.97	0.45-2.06
*PSMA6* and *NOD2/CARD15*						
CC+wild type	—	R	—	—	R	—
CG+GG+mutated	0.60	1.68	0.22-12.58	0.25	3.14	0.43-22.97

R: reference.

**Table 7:** Multivariate Cox analysis in the survival of MM patients.

Variable	Multivariate Cox analysis for OS			Multivariate Cox analysis for PFS		
	*p* value	HR	95% CI	*p* value	HR	95% CI
ISS						
I+II	—	Reference	—	—	Reference	—
III	0.42	1.18	0.77-1.82	0.01	1.57	1.11-2.21
Auto-HSCT						
Yes	—	Reference	—	—	Reference	—
No	0.01	3.82	1.36-10.78	0.14	1.72	0.82-3.61
*PSMA6*						
CC	—	Reference	—	—	Reference	—
CG+GG	0.86	1.42	0.02-82.33	0.41	5.18	0.095-281.82
*NOD2/CARD15*						
Wild type	—	Reference	—	—	Reference	—
Mutated	^∗^	^∗^	^∗^	^∗^	^∗^	^∗^

^∗^Too small group for analysis.

**Table 8:** *NOD2/CARD15* mutation and *PSMA6* polymorphism in the response rate of MM patients.

Variable	Response rate	
	CR+VGPR+PR+SD	PD
	*p* value	OR (95% CI)
ISS		
I+II	—	Reference
III	<0.001	5.44 (2.14-13.84)
Auto-HSCT		
Yes	—	Reference
No	0.015	3.36 (1.22-9.25)
*PSMA6*		
CC	—	Reference
CG+GG	0.05	5.0 (1.07-23.16)
*NOD2/CARD15*		
Wild type	—	Reference
Mutated	0.09	0.4 (0.13-1.18)
*PSMA6* and *NOD2/CARD15*		
CC+wild type	—	Reference
CG+GG+mutated	0.42	^∗^

^∗^Too small group for analysis. CR: complete response; VGPR: very good partial response; PR: partial response; SD: stable disease; PD: progressive disease patients—according to the response criteria for MM [14, 15].

**Table 9:** The clinical values of MM patients included to the study taking into account the studied polymorphisms.

Variables	MM patients	*NOD2/CARD15*			*PSMA6*		
		Wild type	Mutated	*p* value	CC	CG+GG	*p* value
Mean age (years)^∗^	65.32	65.20	65.93	0.78	65.39	65.0	0.87
Free light chain ratio^∗^	303	241.41	617.37	0.08	324.44	217.14	0.59
% of plasma cells in bone marrow^∗^	30.75	31.18	28.40	0.62	30.05	33.88	0.47
Albumins (g/dl)^∗^	3.57	3.58	3.56	0.91	3.53	3.75	0.20
*β*2-Microglobulin^∗^ (mg/l)	5.98	5.79	6.93	0.30	6.26	4.81	0.15
Calcium^∗^ (mM/l)	2.45	2.43	2.53	0.27	2.45	2.43	0.81
Hemoglobin^∗^ (g/dl)	10.38	10.39	10.33	0.91	10.35	10.51	0.74
Creatinine^∗^ (mg/dl)	1.57	1.49	1.98	0.28	1.69	1.07	0.16
C-reactive protein^∗^ (mg/l)	15.68	14.0	23.68	0.31	16.57	12.26	0.63
Estimated glomerular filtration rate^∗^ (ml/min/1.73 m^2^)		70.0	57.99	0.15	66.18	76.19	0.20
PFS (months)	18.32	18.59	16.87	0.73	18.25	18.57	0.94
OS (months)	31	26.94	21.31	0.43	26.24	25.15	0.87

^∗^At diagnosis.

**Table 10:** The effect of different bortezomib doses on bone marrow cell apoptosis, viability, and necrosis (average values).

Genotypes	Bortezomib/DMSO											
	Control 0.1% DMSO (0 nM)	*p* value	1 nM	*p* value	2 nM	*p* value	4 nM	*p* value	8 nM	*p* value	12 nM	*p* value
Apoptotic cells (%)												
Mean	5.45	—	15.44	—	17.49	—	25.55	—	37.52	—	47.68	—
*NOD2/CARD15* wild type	5.10	0.30	15.51	0.92	17.29	0.66	26.18	0.37	39.64	0.018	49.48	0.03
NOD2/CARD15 mutated	6.96		15.10		18.34		22.78		28.28		39.86	
*PSMA6* CC	5.46	0.97	14.79	0.49	17.65	0.76	24.66	0.30	35.58	0.07	47.15	0.61
*PSMA6* CG+GG	5.41		17.39		17.0		28.19		43.33		49.25	
CC+wild type	5.19	0.55	14.46	0.78	17.52	0.84	25.09	0.81	37.07	0.34	48.86	0.45
CG+GG+mutated	6.75		12.97		18.25		23.75		30.77		43.97	
Necrotic cells (%)												
Mean	2.43	—	4.35	—	6.65	—	11.69	—	12.84	—	14.93	—
*NOD2/CARD15* wild type	2.60	0.47	4.38	0.87	6.61	0.91	12.09	0.36	13.15	0.52	15.37	0.31
NOD2/CARD15 mutated	1.68		4.21		6.78		9.97		11.43		13.02	
*PSMA6* CC	2.38	0.85	4.64	0.23	6.66	0.97	11.27	0.42	12.76	0.91	14.99	0.90
*PSMA6* CG+GG	2.58		3.48		6.61		12.94		13.04		14.75	
CC+wild type	2.58	0.93	4.63	0.56	6.67	0.88	11.92	0.66	13.09	0.85	15.46	0.71
CG+GG+mutated	2.42		3.63		7.05		13.37		12.30		14.22	
Viable cells (%)												
Mean	89.17	—	79.58	—	76.08	—	66.18	—	59.71	—	53.32	—
*NOD2/CARD15* wild type	89.56	0.42	78.91	0.40	76.16	0.91	65.62	0.49	58.60	0.14	52.83	0.42
NOD2/CARD15 mutated	87.49		82.47		75.69		68.60		64.51		55.43	
*PSMA6* CC	90.06	0.12	80.65	0.26	75.39	0.48	66.88	0.47	60.69	0.28	53.93	0.40
*PSMA6* CG+GG	86.48		76.35		78.13		64.07		56.76		51.47	
CC+wild type	90.40	0.29	79.99	0.95	75.34	0.95	66.37	0.95	59.71	0.73	53.05	0.61
CG+GG+mutated	86.85		79.61		75.74		66.80		61.67		50.45	

## Data Availability

The clinical data used to support the findings of this study are available from the corresponding author upon request.
